# Contrast-Enhanced EUS for Differential Diagnosis of Pancreatic Masses: A Meta-Analysis

**DOI:** 10.1155/2019/1670183

**Published:** 2019-03-06

**Authors:** Sibin Mei, Mengyu Wang, Leimin Sun

**Affiliations:** ^1^Department of Gastroenterology, Sir Run Run Shaw Hospital, Zhejiang University School of Medicine, Hangzhou 310016, China; ^2^Institute of Gastroenterology, Zhejiang University (IGZJU), Hangzhou 310016, China

## Abstract

**Background:**

Though methods for the diagnosis of pancreatic masses are various, such as ultrasonography (US), computed tomography (CT), endoscopic ultrasonography (EUS), and contrast-enhanced computed tomography (CE-CT), their sensitivity, specificity, and accuracy are not quite satisfying. Contrast-enhanced endoscopic ultrasonography (CE-EUS), as a new technique, has its own unique advantages in diagnosing pancreatic disease. However, its sensitivity, specificity, and accuracy are still controversial.

**Objective:**

To evaluate the accuracy of CE-EUS for differential diagnosis between benign and malignant pancreatic mass lesions.

**Design:**

Eighteen relevant articles systemically searched from PubMed, Web of Science, Ovid, Scopus, and MEDLINE were selected. The pooled results were calculated in a fixed effects model.

**Main Outcome Measurement:**

The pooled sensitivity, specificity, positive likelihood ratio (LR), negative likelihood ratio, diagnostic odds ratio (OR), and summary receiver operating characteristic (SROC) curve.

**Results:**

The pooled sensitivity, specificity, and diagnostic odds ratio of CE-EUS for the differential diagnosis of pancreatic adenocarcinomas were 0.91 (95% confidence interval (CI), 0.89-0.93), 0.86 (95% CI, 0.83-0.89), and 69.50 (95% CI, 48.89-98.80), respectively. The SROC area under the curve was 0.9545. The subgroup analysis based on excluding the outliers showed that the heterogeneity was eliminated and the pooled sensitivity and specificity were 0.92 (95% CI, 0.90-0.93) and 0.87 (95% CI, 0.84-0.89), respectively. The SROC area under the curve was 0.9569.

**Conclusion:**

CE-EUS is a useful method to distinguish pancreatic adenocarcinoma from other pancreatic diseases. Compared with EUS elastography, it has higher specificity. However, it is still not superior to pathological diagnosis for the identification of pancreatic carcinomas.

## 1. Introduction

As the fourth leading cause of cancer-related death in Western countries [1], pancreatic cancer remains a big concern and health burden for both the government and individuals. Due to the lack of early screening methods, patients are often diagnosed with progressive pancreatic cancer on their first visit. Meanwhile, the effectivity rate of currently available treatments, such as chemotherapy, radiotherapy, and surgery, is relatively low [2]. The prognosis of pancreatic cancer is poor, with median survival time ranging from 6 to 10 months [3].

Therefore, to improve the early diagnostic rate of pancreatic cancer, early detection, and differential diagnosis of pancreatic masses are critical. Current methods available for differential diagnosis of pancreatic masses are various, among which EUS, contrast-enhanced ultrasonography (CE-US), CE-CT, CE elastography, endoscopic ultrasonography fine-needle aspiration (EUS-FNA), and CE-EUS are commonly used in recent years.

EUS shows quite high sensitivity due to its high resolution. However, its specificity is relatively low. Tumors, such as pancreatic neuroendocrine carcinoma and inflammatory cystadenoma, are all presented with low-echo crumb images, which are hard to distinguish on EUS.

EUS-FNA is commonly used in clinical settings as the gold standard for pancreatic mass diagnosis. The diagnostic sensitivity and specificity are 80%-85% and nearly 100%, respectively. However, related complications, such as acute pancreatitis, hemorrhage, and infection, may occur. In addition, when patients have concomitant pancreatitis, the false negative rate of pancreatic mass diagnosis using EUS-FNA can increase to 20%-40% [4–6].

CE-EUS is a newly established method which combines the advantage of high-resolution ultrasound of internal organs with the administration of ultrasound contrast agents [7]. It was first applied in pancreatic mass diagnosis in 1995 [8]. As a noninvasive diagnostic technique combining both advantages of EUS and CE, it can further distinguish properties of low-echo pancreatic masses by the density of blood vessels in pancreatic tumor imaging.

Currently, which method is better for pancreatic mass diagnosis remains unclear. Therefore, this meta-analysis was conducted to evaluate the accuracy of CE-EUS and to figure out whether CE-FNA can be replaced by CE-EUS in pancreatic mass diagnosis. The earlier version of this meta-analysis has been presented as an abstract at the 15th Congress of Gastroenterology, China [9]. Here, we presented with the latest and detailed version of this study.

## 2. Materials and Methods

### 2.1. Literature Search

We searched related references in PubMed, Web of Science, Ovid, Scopus, and MEDLINE databases from 1980.01 to 2015.12. The search strategies are “contrast imaging, contrast enhancement, echo enhanced, contrast enhanced, EUS, endoscopic ultrasonography, endoscopic ultrasound, ultrasonography, endosonography, pancreatitis, pancreatic cancer, pancreas, pancreatic mass, pancreatic masses.” We also searched cited references of selected studies to expand our research database. Two authors independently searched and extracted the data, with disagreements being resolved by discussion.

### 2.2. Study Selection

Inclusion criteria are as follows: (1) use of CE-EUS for the diagnosis of solid pancreatic masses, (2) use of a reference standard of EUS-FNA samples, surgical histology, or clinical follow-up of at least 6 months, and (3) available data to construct contingency tables for true positive, true negative, false positive, and false negative results.

Exclusion criteria are as follows: (1) no final diagnosis of pancreatic masses, (2) incomplete data to construct contingency tables, (3) repeated abstracts or articles, (4) reviews, and (5) case reports.

### 2.3. Quality of Studies

Eighteen studies were included in this meta-analysis. The quality of included studies was evaluated using QUADAS, with a total of 14 questions being answered with yes, no, or not clear.

### 2.4. Statistical Methods

We used the extracted data to construct a 2^∗^2 contingency table for each study with 0.5 being added to the contingency tables of the studies with 0. We assessed the accuracy of CE-EUS by calculating the pooled sensitivity, specificity, positive likelihood ratio (LR), negative LR, and diagnostic odds ratios (OR). Pooled results were constructed by using both the Mantel-Haenszel method (fixed effects model) when significant heterogeneity was absent and the DerSimonian-Laird method (random effects model) if otherwise. We used the Cochrane *Q* test to test the heterogeneity among studies. Inconsistency (*I*^2^) expresses the variability attributable to heterogeneity across the studies in the form of a percentage. *I*^2^ > 50% was considered significant for heterogeneity. A summary receiver operating characteristic (SROC) curve was produced. The value of the area under the curve close to 1 indicated a well-validated diagnostic test.

For the studies using both diagnostic tests, sensitivity analyses were conducted by using either one of the two or both. Publication bias was further tested by Deeks' asymmetry test, and a funnel plot of diagnostic log odds ratio versus 1/sqrt (effective sample size) was constructed. The slope coefficient with *P* < 0.05 indicated the presence of publication bias. The pooled sensitivity, specificity, positive LR, negative LR, diagnostic OR, SROC curve, and meta-regression were analyzed by using freeware Meta-DiSc, version 1.4 (Ramon y Cajal Hospital, Madrid, Spain). Publication bias was examined by using Stata MP, version 13.0 (Stata Corporation, College Station, TX).

## 3. Result

### 3.1. Studies

**Figure 1:**
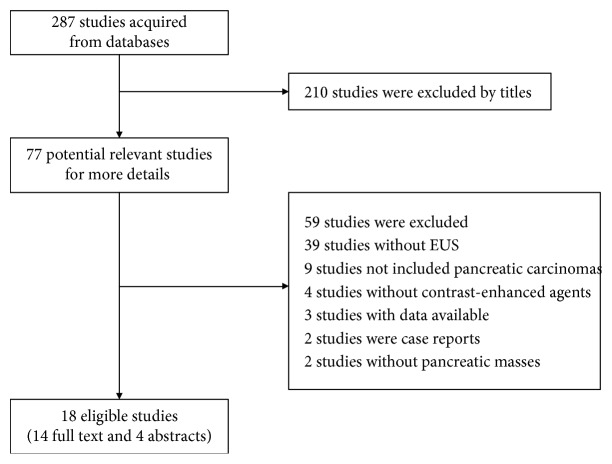
Flow diagram of systematic literature search.

According to the inclusion and exclusion criteria, 18 articles (4 abstracts and 14 full-text articles) were eventually included in this meta-analysis, with a total of 1497 patients involved, as shown in Figure 1 [10–27].

### 3.2. Quality Assessment

Qualities of the eligible studies are assessed according to the QUADAS criteria. The percentage of “yes” of the 18 studies varied from 64.29% to 92.86%. For each item, the percentage varied from 27.78% to 100%.

### 3.3. Meta-Analysis

**Figure 2:**
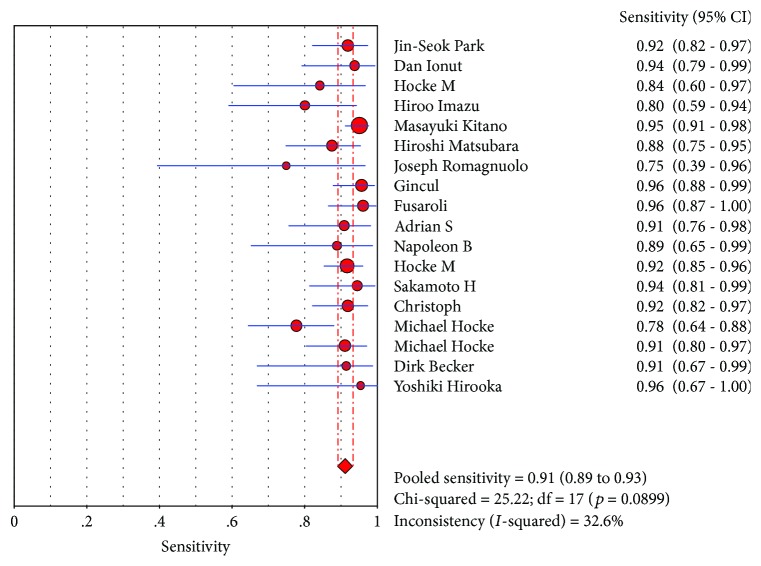
Sensitivity of the selected studies and this meta.

**Figure 3:**
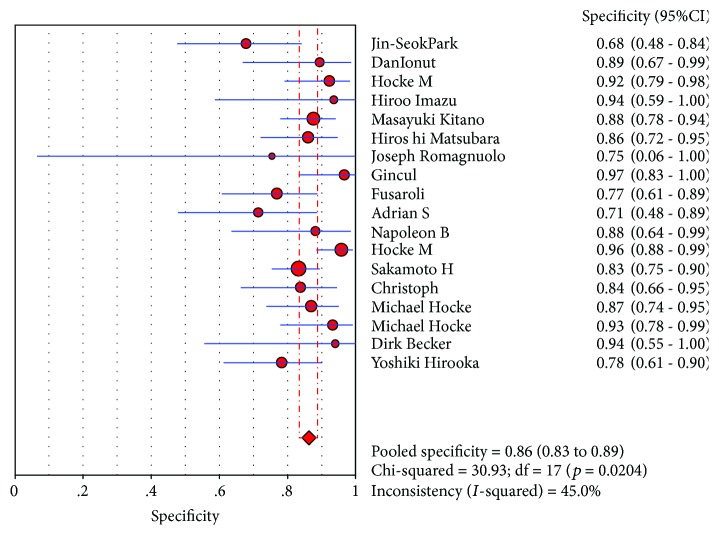
Specificity of the selected studies and this meta.

**Figure 4:**
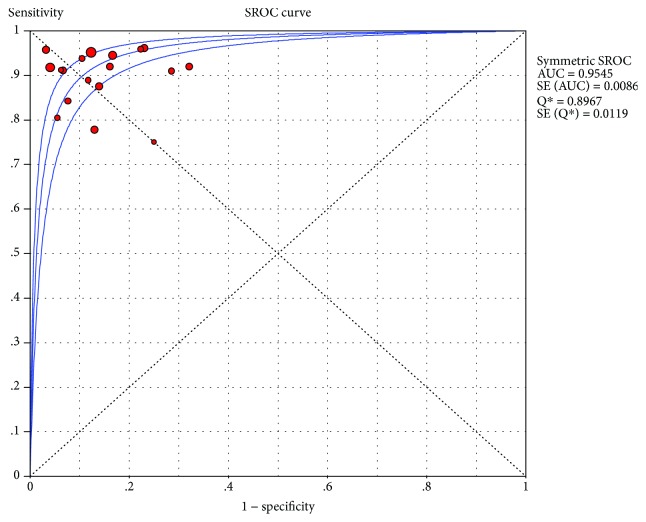
Symmetric SROC of the selected studies and this meta.

**Figure 5:**
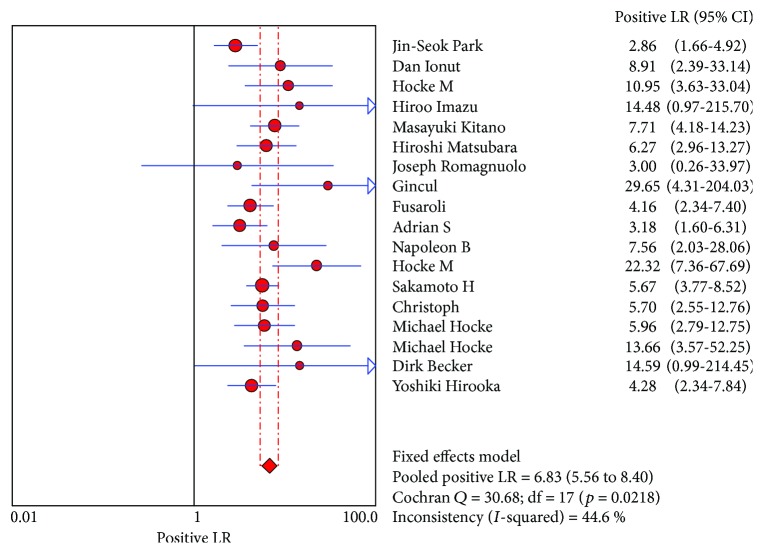
Positive LR of the selected studies and this meta.

**Figure 6:**
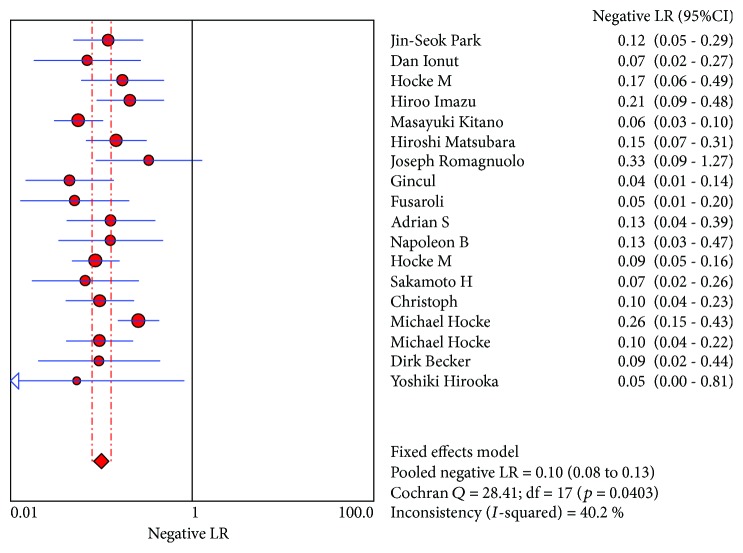
Negative LR of the selected studies and this meta.

**Figure 7:**
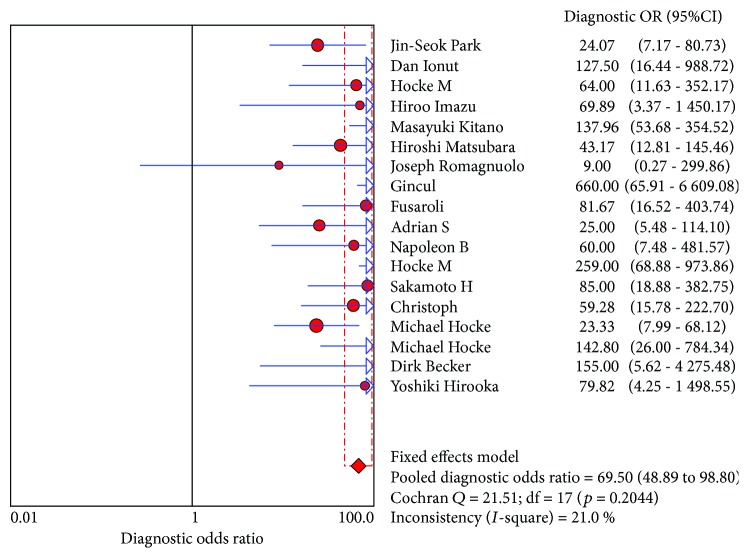
Diagnostic odds ratio of the selected studies and this meta.

CE-EUS had a pooled sensitivity (fixed effects model) of 0.91 (95% CI, 0.89-0.93) in the differential diagnosis of pancreatic adenocarcinomas and other pancreatic focal masses (Figure 2). The pooled specificity (fixed effects model) was 0.86 (95% CI, 0.83-0.89) (Figure 3). Significant heterogeneity was not found neither in sensitivity (Cochran *Q* test = 25.22, df = 17, *P* = 0.0899, *I*^2^ = 32.6%) nor in specificity (Cochran *Q* test = 30.93, df = 17, *P* = 0.0204, *I*^2^ = 45%). The AUC under the SROC was 0.9545 (standard error (SE) = 0.0088) (Figure 4). CE-EUS had a pooled positive LR (fixed effects model) of 6.83 (95% CI, 5.56-8.40) (Figure 5) and a pooled negative LR (fixed effects model) of 0.10 (95% CI, 0.08-0.13) in the diagnosis of pancreatic adenocarcinomas and other pancreatic focal masses (Figure 6). The pooled diagnostic OR was 69.50 (95% CI, 48.89-98.80) (Figure 7). There was no significant heterogeneity neither in positive LR (Cochran *Q* test = 30.68, df = 17, *P* = 0.0218, *I*^2^ = 44.6%) nor in pooled negative LR among the studies (Cochran *Q* test = 28.41, df = 17, *P* = 0.0403, *I*^2^ = 40.2%).

**Figure 8:**
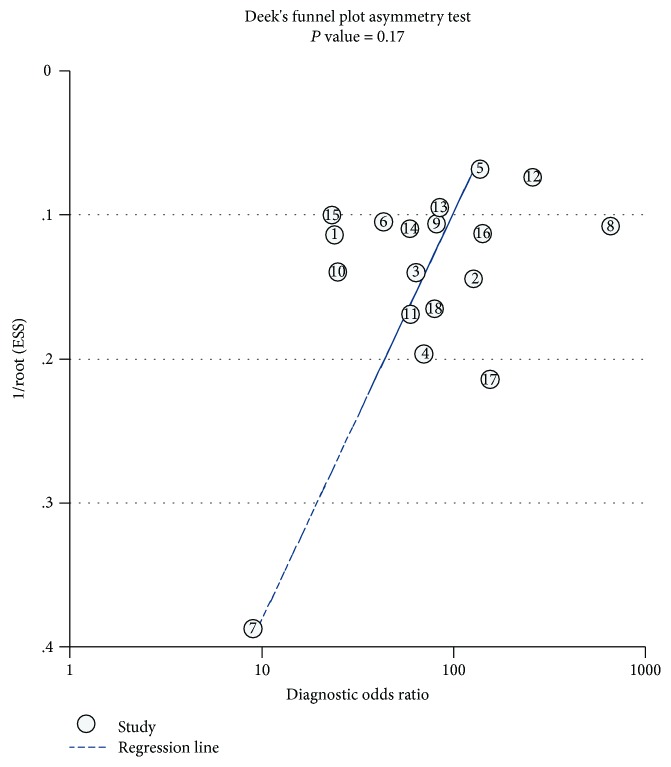
Deeks' funnel plot asymmetry test of the selected studies.

Deeks' funnel plot of diagnostic log odds ratio versus 1/sqrt (effective sample size) did not show significant asymmetry (*P* = 0.17), indicating that there was no significant publication bias in this meta-analysis (Figure 8).

## 4. Discussion

EUS, as a technique applied in diagnosing pancreatic tumors since the 1980s, has attracted increasing attention due to its high-resolution and imaging quality [28]. A great deal of work has reached the conclusion that EUS is more precise compared with traditional abdominal imaging, such as US, CT, and MRI [29–31]. Low-echo conglomeration shadow is the characteristic manifestation of pancreatic tumors with quite high sensitivity and specificity. But not all the low-echo conglomerations are pancreatic adenocarcinomas. Pancreatic neuroendocrine tumor, pancreatic cyst, and chronic pancreas inflammatory cystadenoma are all presented with the image of low-echo conglomeration on the EUS. Therefore, further differentiation between these masses is necessary.

As one of the gold standards of diagnosing pancreatic tumors, EUS-FNA has a specificity of nearly 100% through accessing the pathological diagnosis directly. However, its high false negative rate of 20%-40%, especially when it comes to diagnosis of pancreatic tumors combined with chronic pancreatitis, limits its clinical application. CE elastography is a new diagnosis technique which has gained significant effects in clinical settings recently. A meta-analysis investigating CE elastography showed that it has a high sensitivity of 0.95 in the diagnosis of benign and malignant pancreatic tumors but the specificity is only 0.67 [32]. Thus, CE elastography can hardly take the place of CE-FNA if its specificity could not be improved in the future.

In comparison, CE-EUS owns different mechanisms and shows relatively higher diagnostic accuracy. Contrast agents used in EUS are gas-containing microbubbles, which would oscillate and generate an acoustic signal when hit by an ultrasonic wave [33]. Therefore, apart from assessing the echogenicity of pancreatic masses themselves, CE-EUS could assess their vascularity by detecting the acoustic signal after intravenous bolus injection of contrast agents. Through demarcating vascular landmarks, detecting vascular obliteration by a thrombus or tumor, and examining microvascular blood flow to organs and lesions [34], more information can be gained for further differentiation.

Based on the mechanical index (MI) displayed on the monitor to indicate the acoustic power, CE-EUS can be further divided into contrast-enhanced high-mechanical index endoscopic ultrasonography (CEHMI-EUS) and contrast-enhanced low-mechanical index endoscopic ultrasonography (CELMI-EUS) [35]. CEHMI-EUS is commonly used with the first-generation contrast enhancers, which requires microbubble destruction and provides only scattered scanning of the lesion [26], whereas CELMI-EUS is applied with the new contrast enhancers, providing clinicians with real-time viewing of the contrast enhancer effects [26].

Currently, ultrasound contrast agents have already developed to the third generation. SonoVue and Sonazoid are most commonly used in clinical settings, and they both belong to the second generation [33]. In this meta-analysis, SonoVue was used in 7 studies and Sonazoid was used in 4 studies as the contrast enhancers. Both are excreted through the respiratory system; therefore, CE-EUS can also be applied to patients with liver and renal dysfunction. Several studies have shown that the ultrasound contrast agents are safe and effective and have no obvious long-term adverse effects [36, 37].

Differential diagnosis of pancreatic masses can be achieved based on their CE-EUS imaging findings. In CE-EUS, diffuse homogeneous enhancement is presented in normal pancreatic parenchyma and the normal bile duct and pancreatic duct are depicted as nonenhanced ductal structures [38]. Pancreatic adenocarcinomas are typically hypoenhanced [39]. Within the tumor, arterial vessel architecture is irregular and venous vessels are absent [40]. In comparison, focal chronic pancreatitis is commonly hyperenhanced with regular arterial microvascular architecture [40]. Besides, both venous and arterial vessels are presented in chronic pancreatitis [40]. Small singular well-demarcated lesion with hyperenhancement strongly indicates neuroendocrine tumors of the pancreas [40]. For pancreatic cystic lesions, in general, wall and nodule vascularization indicates a cystic tumor which cannot be seen in dysontogenetic cysts or pseudocysts [41]. Within cystic tumors, typical serous cystadenoma shows the intralesional septation enhancement [42]. In comparison, mucinous cystadenoma is characterized by intralesional irregular septation and parietal nodule enhancement [42]. Malignancy of intraductal papillary mucinous neoplasms (IPMNs) can be assessed according to the enhancement pattern of mural nodules. Papillary module and invasive nodule are evidences of invasive IPMNs while low papillary nodule and polypoid nodule indicate benign IPMNs [38]. Solid pseudopapillary neoplasms (SPNs) are presented with inhomogeneous enhancement of the thickened peripheral capsule and solid components surrounding the cystic and necrotic avascular areas [42].

This meta-analysis shows that CE-EUS has a high sensitivity of 0.92 (95% CI, 0.90-0.93) and a relatively high specificity of 0.86 (95% CI, 0.84-0.89) in the diagnosis of benign and malignant pancreatic tumors. There is no obvious heterogeneity between studies. The Spearman correlation coefficient between the log of sensitivity and log of 1−specificity was 0.089 (*P* = 0.751), which shows no obvious threshold effect between studies. Meta-regression analysis also finds no possible sources of heterogeneity. The funnel chart shows no publication bias. We exclude the articles from both Park et al. [14] and Romagnuolo et al. [27] according to the summary of the forest figure. Subgroup analysis shows that pooled calculation values increase.

## 5. Conclusions

Taken together, it is still difficult for CE-EUS to replace CE-FNA in making a definite diagnosis. However, considering its higher accuracy compared with CT and noninvasive characteristic compared with CE-FNA, CE-EUS should be an important part in the pancreatic mass diagnosis algorithm. For patients clinically suspicious of pancreatic mass, helical CT should be firstly conducted. If patients are presented with negative CT results but still strongly suspicious of pancreatic mass or patients are presented with ambiguous CT results and need further confirmation, CE-EUS should be the choice. Based on the result of CE-EUS, clinicians can decide whether CE-FNA is needed or not.

In the future, the accumulation of operating experience, the establishment of a more accurate diagnosis standard, the development of CE-EUS technologies, and a larger amount of summary data analysis may help to improve the diagnostic accuracy in the diagnosis between benign and malignant pancreatic tumors using CE-EUS. Which is better between CE-EUS and CE-FNA for pancreatic mass diagnosis will be an interesting debate at that time.

## Data Availability

The data used to support the findings of this study are included within the article.
